# Investigation of Piezoelectricity and Resistivity of Surface Modified Barium Titanate Nanocomposites

**DOI:** 10.3390/polym11122123

**Published:** 2019-12-17

**Authors:** Udhay Sundar, Zichen Lao, Kimberly Cook-Chennault

**Affiliations:** 1 Portland Technology Development, Intel Corporation, Portland, OR 97124, USA; udhay.sundar@gmail.com; 2 Mechanical Engineering and Applied Mechanics, University of Pennsylvania, Philadelphia, PA 19104, USA; lao0910@seas.upenn.edu; 3 Mechanical and Aerospace Engineering Department, Rutgers, the State University of New Jersey, Piscataway, NJ 08854, USA

**Keywords:** piezoelectric, composite, surface treatment, core-shell processing

## Abstract

Polymer-ceramic nanocomposite piezoelectric and dielectric films are of interest because of their possible application to advanced embedded energy storage devices for printed wired electrical boards. The incompatibility of the two constituent materials; hydrophilic ceramic filler, and hydrophobic epoxy limit the filler concentration, and thus, their piezoelectric properties. This work aims to understand the role of surfactant concentration in establishing meaningful interfacial layers between the epoxy and ceramic filler particles by observing particle surface morphology, piezoelectric strain coefficients, and resistivity spectra. A comprehensive study of nanocomposites, comprising non-treated and surface treated barium titanate (BTO), embedded within an epoxy matrix, was performed. The surface treatments were performed with two types of coupling agents: Ethanol and 3-glycidyloxypropyltrimethoxysilan. The observations of particle agglomeration, piezoelectric strain coefficients, and resistivity were compared, where the most ideal properties were found for concentrations of 0.02 and 0.025. This work demonstrates that the interfacial core-shell processing layer concentration influences the macroscopic properties of nanocomposites, and the opportunities for tuning interfacial layers for desirable characteristics of specific applications.

## 1. Introduction

Piezoelectric materials are primarily used as transducers to convert mechanical energy into electrical energy, or vice versa. To this end, they are employed in structural health monitoring applications for civil structures [1,2,3,4], industrial sensors and actuators [5,6,7], bioengineered scaffolds [8,9], energy storage applications [10,11], embedded capacitor materials [12,13,14,15], and vibration-based energy harvesting applications, due to their ability to convert mechanical energy to electrical energy [5,16,17]. Research and innovation in piezoelectric and dielectric materials is also motivated by the Internet of Things (IoT) [5,10,16]—namely devices, vehicles, buildings, that are wirelessly connected via electronics, software, sensors, and network connectivity. The realization of devices, such as these, that can transmit information, communicate wirelessly, and self-power themselves requires the development and optimization of advanced piezoelectric and dielectric materials. Also, systems such as these that employ sensors, are powered using conventional batteries, which limit their operational cycle. Replacement of the batteries can prove problematic in cases where the batteries are in remote locations, such as structural sensors on a bridge or as tracking devices on moving objects [18]. Hence, it is desirable to use self-powered sensors in such applications. Piezoelectric energy harvesting devices provide an alternative to traditional battery powered systems, where they can be used to self-power themselves and other components within micro-electromechanical systems (MES), where they can be integrated into hybrid power systems [5,19,20].

For many of the aforementioned applications, either ceramic piezoelectric and dielectric oxides or polymers are used, where the ceramic oxides possess high piezoelectric strain coefficients, permittivity, and stiffness values. These materials, however, also suffer from high dielectric loss over broad bands of frequency and relatively high mechanical stiffness, which makes them susceptible to premature failure during cyclic operation, and inherently low breakdown field strength, which limits their available energy density for energy storage and IOT applications. When these materials are used as active materials for capacitors, they can experience brittle fracture behavior from defects, such as cracks to the electrical degradation and mechanical failure [21,22].

On the other hand, polymeric piezoelectric and dielectric materials have high breakdown field strengths, low dielectric losses, and are inherently ductile, which makes them easy to process into various shapes and films, and easier to three-dimensional (3D) print. However, these materials have very low dielectric constants and low piezoelectric strain coefficients. Hence, polymer-ceramic composites have garnered interest as embedded capacitor materials because of their ease of fabrication, compatibility with printed wiring board (PWB) technology, and high relative permittivity (5–20,000) [23,24,25,26]. Workers have demonstrated that the dielectric constants of polymer-ceramic composites could be enhanced by adding conductive particles in insulative polymeric matrices as a third phase in the composite [27,28,29,30,31], in order to enhance overall piezoelectric strain coefficients and permittivity of the composite. However, these composites suffer from high dielectric losses due to increased dielectric loss, low breakdown strengths, and current leakage values, which make them less amenable to electrical polarization [32,33,34] and likely to possess relatively low piezoelectric strain coefficients.

The 0–3 connectivity piezoelectric composites have been extensively studied [6,35], where emphasis has primarily focused on PZT-based composites over the last several decades [36,37,38,39] and lead-free alternatives, such as barium titanate (BaTiO_3_)-based composites most recently [11,29,40]. Yamamoto et al. [41] was one of the first to examine the influence of powder particle size in the fabrication of barium titanate (BTO)—polymer composites. In this work, it was found conjectured that, BTO mean particle diameters less than 0.1 µm were nearly zero, which was attributed to the pseudo-cubic phase of the BTO powder. While, BTO powder above 0.3 µm was found to be in the tetragonal phase, and therefore, able to render higher values of piezoelectric strain (~1.0 pC/N). Later, others such as, Ehterami et al. [9] fabricated porous BTO-based composite bone scaffolds comprising BTO micron-scaled powder and nano-scaled hydroxyapatite. In this work, piezoelectric properties were influenced by the polarization time, electric field intensity, and sintering time of the scaffolds, where samples comprising 50% BTO by wt % possessed d_33_ values that ranged from ~0.25 pC/N to 4.5 pC/N depending on the aforementioned processing parameters. Similarly, Capsal et al. [42] examined the longitudinal piezoelectric strain coefficient of BTO-Polyamide 11 nanocomposites as a function of BTO particle mean diameter (700, 300, 100, and 50 nm) and polarization temperature, time, and electrical field intensity. In this work, composites with larger mean diameters rendered higher piezoelectric strain coefficients, i.e., samples with 50 nm mean diameters rendered no piezoelectric strain coefficients, while those with 100, 300, and 700 nm rendered maximum d_33_ values of ~1, ~4.5 and 5 pC/N. They noted that the ferroelectric/paraelectric transition was not observed for 50 and 100 nm particles. However, the cubic to tetragonal transition seemed to take place between the BTO particle size of 100 to 300 nm. Also, workers such as, Defebvin et al. [43] fabricated nano-composites that incorporated nano BTO powder (particle mean diameter ~200 nm) at concentrations of 10, 20, and 30 wt % BTO within a poly(-vinylidene fluoride) (PVDF) matrix, where the maximum piezoelectric strain coefficient of ~1.5 pC/N was recorded for 30 wt % of BTO. Similarly, [44] fabricated PVDF (mean diameter ~3 µm)-BTO (particle mean diameter ~1 µm) composites and determined that the piezoelectric properties of the composites were influenced by the calcining temperature of the BTO, and size and morphology of the BTO particle. Li et al. [44] also concluded that sintering the BTO powder at higher temperatures up to ~950 °C enabled the BTO to transition from the rhombohedral to the tetragonal phase, while sintering temperatures above this reduced the appearance of the (002) peak.

The composites with filler loadings greater than 50% by volume fraction have been shown to exhibit higher overall piezoelectric and permittivity values, where high piezoelectric strain coefficients have been associated with higher mean diameters of inclusions. Nanoparticles in polymer matrices have several advantages, such as increased dielectric breakdown strength, improved voltage endurance over the life of the sample and by suppressing space charge and enhancing the partial discharge resistance of dielectric polymers [45]. However, the tendency for nanoparticles to agglomerate prior to, and during, the processing of nanocomposites within the matrix [46,47] presents an obstacle for reliable properties. Agglomeration within nanocomposites is typically attributed to two main factors. The first is the high degree of surface area to volume ratios, which have high surface energy, and causes them to form bonds with one another to diminish this energy. The second factor is the van der Waals forces that arise between all molecules and particles. Uncoated nanoparticles of metals, metal oxides and ceramic materials have strong van der Waals forces [46,47]. Surfactants and coupling agents can reduce this surface energy by forming covalent bonds with the nanoparticles, thereby, reducing the inter-particle agglomeration.

Van der Waals forces exist between all molecules and particles, in air, vacuum, and liquid. The formation of aggregates can be prevented by steric or electrostatic stabilization. Steric stabilization usually involves coating the nanoparticles with a tightly bound polymer or surfactant monolayer. Electrostatic stabilization can be used in aqueous solutions where the particles become charged and/or encapsulated by a hydration layer, which further prevents their agglomeration [46].

In addition to the agglomerations of nanoparticles, the inherent incompatibility of inorganic-organic constituent materials, that make up a composite, creates further difficulties in processing [24,25,48,49,50,51]. In BaTiO_3_/epoxy composites, the surface of BaTiO_3_ particles have residual hydroxyl groups, which are hydrophilic in nature, while the epoxy resin and organic solvent are hydrophobic. Hence, BaTiO_3_ tends to agglomerate and separate from the organic solvent or resin, resulting in processing difficulties, as discussed previously [24,25,52]. As a result, dielectric polymer nano-composites, comprised of high volume fractions of ceramic fillers, have received limited attention due to the difficulties in processing and particle dispersion.

Identification of a suitable surface modification method could address both the aforementioned problems. Researchers have looked at modifying BaTiO_3_ by treating it with hydrogen peroxide (H_2_O_2_) to further hydroxylate the surface [53,54,55,56,57]. To improve the compatibility between inorganic fillers and polymer materials, surface modifiers have been used, such as macromolecular surface modifiers [58,59] and small-molecular weight ones, such as surface active agents, e.g., silane coupling agents and titanate coupling agents [60,61,62,63]. Surface modification with a coupling agent, which has two functional groups, reacting with either, BaTiO_3_ or epoxy by forming covalent bonds. Silane coupling agents were studied in dielectric polymer composites that did not include BaTiO_3_, such as epoxy/aluminum [64] and epoxy/fosterite (Mg_2_SiO_4_) [65]. For example, Zhou et al. [64] investigated the influence of coupling agents by modifying the surface of aluminum (Al) particles by examining their microstructure and dielectric properties. In this study, the coupling agents (silane KH550 and silane KH560) were used to improve the interfacial bond strength between the aluminum and epoxy in Al/epoxy composites. The dielectric analysis of the composites at 70 wt % of Al particles yielded increased permittivity values, 34 for the treated particles, as opposed to the pristine ones, which had permittivity equal 19.6. Surface-treated particles also exhibited reduced dielectric loss values. The use of coupling agents improved the interfacial bonding strength between the aluminum particles and the epoxy resin decreased the voids and defects at the phase interfaces. Dang et al. [66] studied high dielectric permittivity BaTiO_3_/epoxy composites with different size BaTiO_3_ particles i.e., 0.7 µm and 0.1 µm. The epoxy resin (DER 661) was used as the polymer matrix. It was reported that KH550 improved the interaction between the epoxy resin and BaTiO_3_ particles, and therefore, led to homogeneous dispersion. This also led to increased dielectric permittivity (~26 for the untreated composite to ~28 for the surface treated composite at 10 kHz) and decreased dielectric loss values (from 0.08 for the untreated composite to 0.06 for the surface treated composite). It is evident that the size of BaTiO_3_ has an effect on the dielectric property of the composites. Similarly, researchers, such as Ramesh et al. [52], organically modified BaTiO_3_ nanoparticles prior to embedding them into an epoxy resin matrix (EPON 828) and found that the dielectric permittivity could be enhanced with core-shell processing of the nano-filler. While, several researchers have investigated the role of core-shell processing on dielectric properties of nanocomposites, less has been explored regarding the role of filler core-shell processing and its concentration on the piezoelectric and resistive properties of nanocomposite films.

The goal of this work is to understand how surface modification of the active nanoparticle can influence the particle-matrix interface and ultimately the piezoelectric and resistive properties of the nanocomposite films. Hence, the effect of the surface modification of the BaTiO_3_ nanoparticles using ethanol and a silane coupling agent will be studied and the optimum concentration of silane coupling agent will be identified. The composite interfacial properties and morphology have been studied with the aid of scanning electron microscopy (SEM) and energy dispersive X-ray spectroscopy (EDS) micrograph images. In order to understand the influence of surface modification of BaTiO_3_ on interfacial properties formed in the composite, the piezoelectric and resistivity properties of the BaTiO_3_/epoxy nanocomposite are described and correlated to the surface processing technique and concentration of the coupling agent. In this work, BaTiO_3_/epoxy thick film composites were fabricated where the volume fractions of BaTiO_3_ were varied from 0.10 to 0.60. A minimum of four samples of each volume fraction was fabricated for the studies described herein. Nano-sized barium titanate (BaTiO_3_) powder was surface modified using ethanol (3 mL per unit gram of BaTiO_3_) and varying concentrations (in terms of volume fraction of the combined mixture) of silane coupling agent (0.01, 0.015, 0.020, and 0.025).

## 2. Materials and Methods

### 2.1. Materials

The materials used were BaTiO_3_ (Sigma-Aldrich, <100 nm particle size, ≥99%, St. Louis, MO, USA) in the cubic crystalline phase, Epofix Cold-Setting embedding resin (Bisphenol-A-Diglycidylether, Electron Microscopy Sciences) [67], Triethylenetetramine (epoxy hardener) and 3-Glycidyloxypropyltrimethoxysilane (Sigma-Aldrich, ≥98%, KH-560). The dielectric and physical properties of the constituent materials: BaTiO_3_, DGEBA epoxy and KH-560 are presented in Table 1, Table 2 and Table 3 respectively.

### 2.2. Experimental Method

#### 2.2.1. Surface Modification of BTO with Ethanol

The surface modification of the BaTiO_3_ nano-powder using ethanol was used as the baseline datum for all studies, where it was used as a dispersant to aid in breaking down the particle-particle agglomerations [31]. Hence, similar to other workers [31], a time study was carried out to determine the minimum amount of time required to break down the majority of the agglomerations, where the mixture containing BaTiO_3_ and ethanol was placed in an ultrasonicator between 1 and 5 h, and the microstructure observed every hour. A paraffin film was placed at the top of the beaker to prevent evaporation of the ethanol into the air. The BaTiO_3_ particle morphology and distribution of the nanoparticles were observed with the aid of a Zeiss Sigma Field Emission scanning electron microscope (SEM, Oberkochen, Germany) and an Oxford INCA PentaFET x3 8100 energy dispersive X-ray spectroscopy (EDS, Oberkochen, Germany). The SEM micrograph images were captured of the BaTiO_3_ as received and after surface modification at each time increment, i.e., every hour for five hours.

#### 2.2.2. Surface Modification of BTO with Silane Coupling Agent

**Figure 1:**
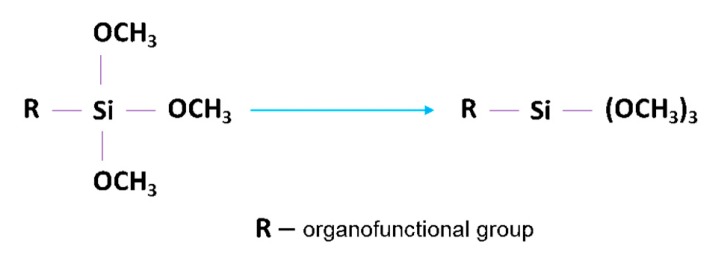
General structure of a silane coupling agent, where R is an organic functional group.

It is well-known that coupling agents improve the adhesion between mineral fillers and organic resin matrices [70], where the coupling agents improve the chemical resistance of the bond across the interface between the resin matrix and mineral filler. In general, coupling agents comprise two different functional groups, where each one reacts with each part of the repulsive system, thereby, promoting adhesion [70]. Silane coupling agents consist of two functional groups, one which can react with the surface of the BaTiO_3_ nanoparticle by forming a covalent bond with the residual hydroxyl groups on its surface, and the other which can react with the epoxy chain of the resin as shown in Figure 1.

In this work, the *glycidyloxypropyl* type silane was chosen as the organic functional group because glycidylfunctional epoxy resin was being employed, i.e., dyglycidyl ether of bisphenol A (DGEBA) is the epoxy used as the matrix materials for the composites [70,71]. Four different concentrations, in terms of volume fraction of the entire mixture, of coupling agent were used, namely, 0.01, 0.015, 0.020, and 0.025 (*v*/*v* i.e., volume fraction percentage) to modify the BaTiO_3_ nanoparticles.

**Figure 2:**
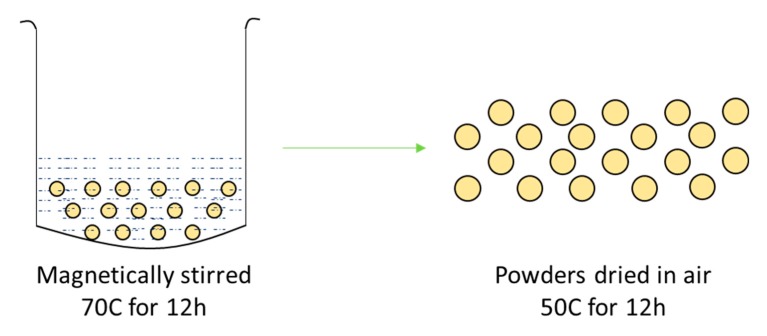
Surface treatment of BaTiO_3_ nanoparticles with KH-560. The mixture was magnetically stirred for 12 h and 70 °C followed by drying in air at 50 °C for an additional 12 h.

For the 0.01 KH-560 treatment, 5 g of BaTiO_3_, 50 mL of ethanol and 0.5 g of KH-560 were mixed in a beaker (amount of KH-560 was varied accordingly for the 0.015, 0.020 and 0.025 treated samples). The mixture was subsequently magnetically stirred for 12 h and 70 °C. The powders were then dried at 50 °C for an additional 12 h in air for the residual ethanol to evaporate. Once the powders were surface-treated, they were then used for sample preparation. The surface modification procedure is shown in Figure 2 and the variation in silane coupling agent concentration is provided in Table 4.

### 2.3. Sol Gel Synthesis and Film Deposition

**Figure 3:**
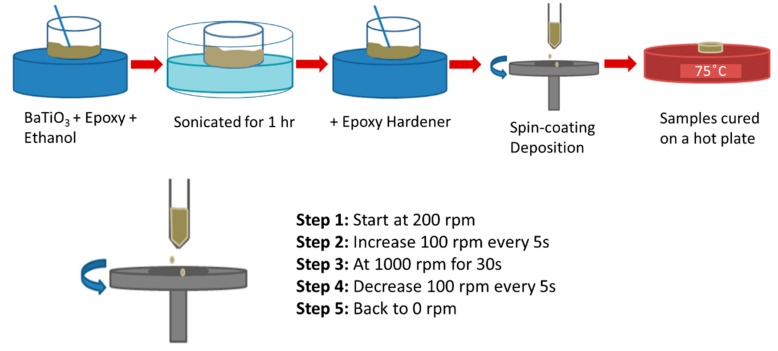
A Schematic diagram of the composite preparation is provided. The contents, i.e., surface-treated BaTiO_3_, epoxy was mixed in a beaker and ultra-sonicated for 1 h, and the subsequent mixture was spin-coated using a multi-step method to ensure uniform dispersion by accelerating and decelerating gradually.

BaTiO_3_/epoxy sol gel (comprising BTO, epoxy with ethanol, or silane coupling agent) was deposited on to stainless steel substrates using a multi-step spin coat and deposition technique. The stainless-steel substrates were 20 mm by 20 mm squares and 0.0254 mm thick. An overview of the fabrication process for the two-phase or diphasic composites is provided in Figure 3. The volume fractions of the BaTiO_3_ were varied from 0.10 to 0.60 (*v*/*v*). The surface-modified nanoparticle (either by ethanol alone or silane coupling agent) are allowed to dry. The dried powder was then weighed to achieve the desired volume fraction and transferred to a beaker. The appropriate amount (based on desired volume fraction) of DGEBA epoxy resin was then added to this beaker by means of a measuring syringe. The subsequently formed mixture was ultra-sonicated for 1 h, whilst being hand-stirred every 15 min.

Finally, the epoxy hardener was added to this mixture. The contents of this combined mixture were transferred via a dropper to the stainless steel substrate, which sits on the spin-coater. The multi-step spin coating method comprised of many steps to ensure that the final speed was achieved in a step-wise manner. The initial speed was set to 200 rpm and increased by 100 rpm every 5 s until it reached 1000 rpm. The substrate spun at 1000 rpm for 30 s before the speed was reduced to 200 rpm in the same way, to ensure that there was no sudden cessation. This spin-coat process was designed to reduce any inertia that may act on the substrate. Once the samples were coated, they were removed and placed on a hot plate in air for 8 h at 75 °C. The samples were then cooled for 24 h. The BaTiO_3_/epoxy composites were corona polarized at 30 kV/mm in air at 75 °C for 30 min using a Spellman SL 1200 high voltage power source.

### 2.4. Film Characterization

The piezoelectric strain coefficient, *d*_33_ was measured using PIEZOTEST’s PiezoMeter PM 300 System. This PiezoTest system can piezoelectric strain coefficients *d*_33_ and *d*_31_. The system works by clamping the sample and subjecting it to a low frequency force of 0.25 N at 110 Hz. The HP 4194A impedance analyzer was used to measure the impedance, *Z*. The real part of impedance is resistance, *R* and the imaginary part is reactance, *X*. The imaginary part physically represents the phase angle between voltage and current, in an ideal capacitor, where the voltage lags behind the current by 90°.

(1) Z=R+jX.

The real part of impedance, i.e., resistance was then used to calculate resistivity, so as to normalize it for units of length as can be seen in Equation (2),

(2) $$\rho =\frac{RA}{t}.$$

Similarly, the admittance, *Y*, is also measured by the HP 4194A impedance analyzer which like impedance is also a complex quantity as shown in (3),

(3) Y=G+jB,

where, *G* is conductance and *B* is susceptance.

## 3. Results

This section may be divided by subheadings. It should provide a concise and precise description of the experimental results, their interpretation as well as the experimental conclusions that can be drawn. The Results and Discussion section is divided into three subsections: (1) Morphological Characterization (BTO-Ethanol Surface Treatment and BTO-Silane Coupling Agent); (2) Piezoelectric Strain Characterization (BTO-Ethanol and BTO-Silane Coupling Agent) and (3) Resistivity (BTO-Ethanol and BTO-Silane Coupling Agent). The Morphological Characterization section provides qualitative observations from the SEM and EDS micrograph images, particle mean diameter of the datum (non-surface treated) samples, ethanol treated and silane coupling agent treated BaTiO_3_ nanoparticles. The Piezoelectric Strain Characterization section elucidates the impact of surface modification on nano-powders on the ability of the materials to store and dissipate charge via dielectric permittivity spectra and dissipation spectra measurements. The Electrical Resistivity section describes the impedance/resistivity characteristics of the nanocomposites.

### 3.1. Morphological Characterization

#### 3.1.1. Morphological Characterization—Surface Treatment with Ethanol—Datum

**Figure 4:**
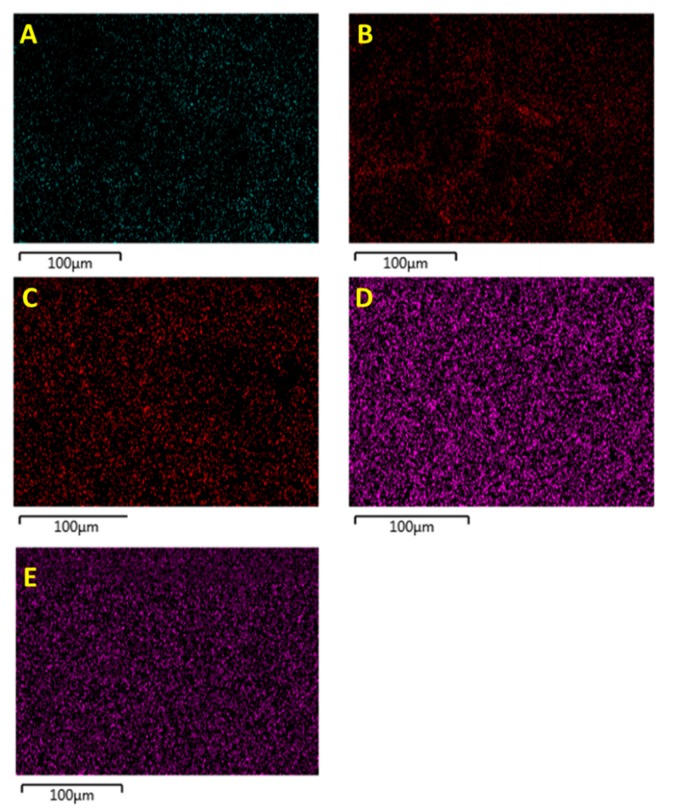
EDS micrograph images of the BT powder surface treated using ethanol for (**A**) 1 h, (**B**) 2 h, (**C**) 3 h, (**D**) 4 h and (**E**) 5 h. It is evident that there is better particle dispersion with longer sonication time in ethanol. However, the optimal sonication time based on the images is for four hours. (Note: The different colors all represent BT nanoparticles, but since they are on different SEM studs, the machine treats them as different sample sets, and hence the change in color).

The non-surface treated BaTiO_3_ composites were used as the datum for this work and compared with the composites that were prepared using ethanol surface treated and silane coupling agent treated BaTiO_3_ nanoparticles. The BaTiO_3_ nanoparticles were surface modified in ethanol at different durations, 1, 2, 3, 4 and 5 h and the dispersion of the particles were studied using EDS micrograph images, as shown in Figure 4.

**Figure 5:**
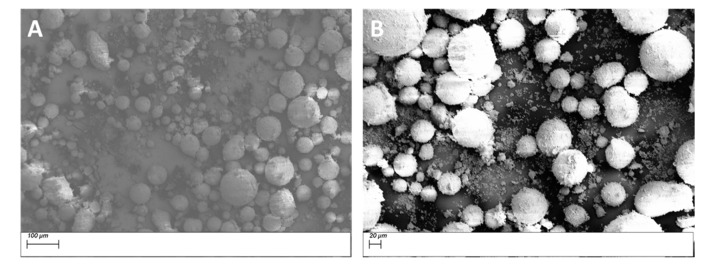
SEM micrograph images of BaTiO_3_ nanoparticles that were surface treated in ethanol for 4 h (**A**) at a scale of 100 µm and (**B**) at a scale of 20 µm. From the images we can see charging of the samples even after applying a gold coating of 30 nm, which could be attributed to the surface charges produced on the BaTiO_3_ nanoparticle due to its reaction with ethanol. This electrostatic charge keeps the BaTiO_3_ nanoparticles from agglomerating as can be seen by the reduction in mean particle size i.e., from ~60 µm to 40 µm.

The EDS images also indicate that the exposure of the BaTiO_3_ nanoparticles to ethanol over extended periods of time diminished the agglomeration and particle-particle clusters. From these results, 4 h was chosen as the ideal surface treatment time for the BaTiO_3_ nanoparticles. From the SEM micrograph images in Figure 5 we can see charging of the samples even after applying a gold coating of 30 nm, which could be attributed to the surface charges produced on the BaTiO_3_ nanoparticle due to its reaction with ethanol. This electrostatic charge keeps the BaTiO_3_ nanoparticles from agglomerating as can be seen by the reduction in mean particle size compared with the untreated nanoparticles (from ~60 µm to 40 µm).

The stability of ceramic particles is based on the summation of the van der Waals attractive forces, electrostatic repulsion and possible steric hindrance from adsorbed surfactants or coupling agents [62,63]. Either electrostatic repulsion or steric hindrance counter acts the strong van der Waals forces to reduce the formation of particle clusters. Ethanol is considered to be a weakly acidic solvent (pK_a_~16 and pH ~<7). As result of this solvent’s polarity a surface charge may be developed on the surface of the ceramic particle (Silica fillers and Calcium Carbonate, CaCO_3_) [72,73] as they are exposed to the solvent over time. Fowkes et al. [74,75] observed the development of a positive surface charge on ceramic particles after they were mixed with ethanol. The surface charge was achieved by proton exchange, i.e., the acidic nature of the adsorbate results in proton transfer to the particle surface. This charged surface that forms on the particles results in electrostatic repulsion between successive particles thereby counter acting the van der Waals attractive forces leading to improved particle dispersions within the organic medium [62].

#### 3.1.2. Morphological Characterization—Surface Treatment with Silane Coupling Agent (KH 560)

**Figure 6:**
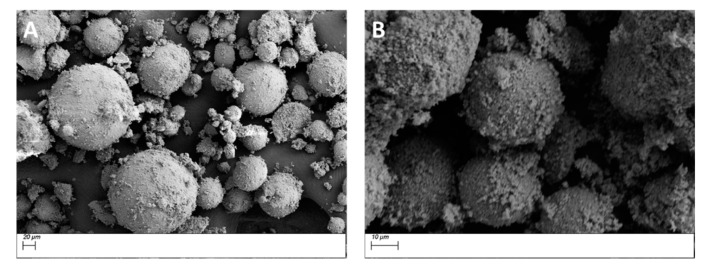
SEM micrograph images of (**A**) pristine BaTiO_3_ particles and (**B**) BaTiO_3_ particles surface treated using SCA 0.01. The images represent the cluster sizes of the of BaTiO_3_ nanoparticles on a carbon tape. The average cluster size of the pristine BaTiO_3_ particles is ~60 µm and that of the BaTiO_3_ particles surface treated using SCA 0.01 is ~30 µm.

**Figure 7:**
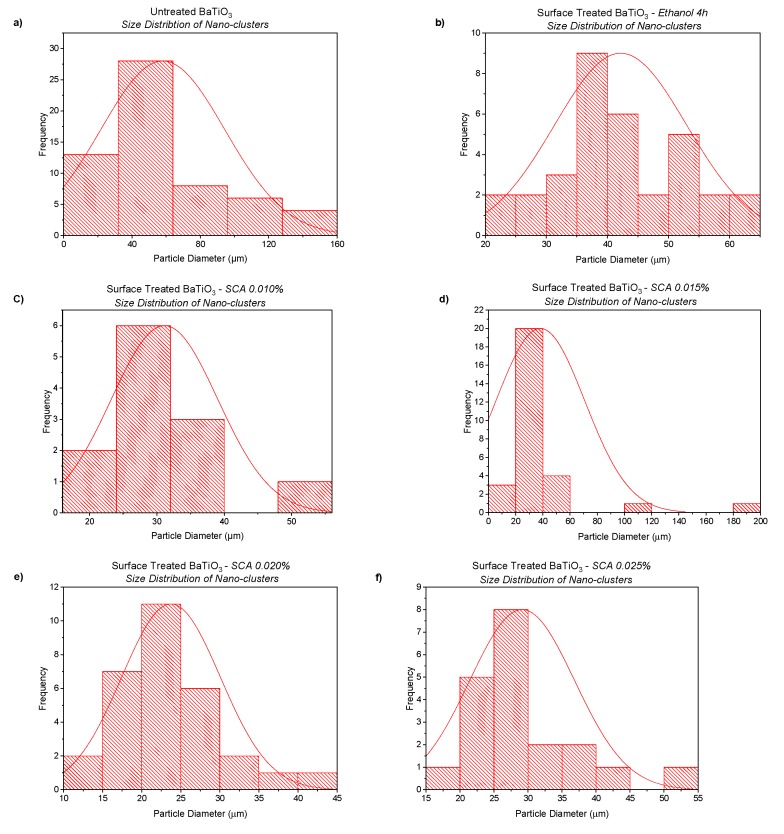
Particle distributions of (**a**) pristine BaTiO_3_ which shows an average cluster size of ~60 µm, (**b**) ethanol surface treated for 4 h with an average cliuster size of ~40 µm, (**c**) 0.01 silane coupling agent treated BaTiO_3_ with an average cluster size of ~30 µm. It can observed that the agglomerates reduce with surface treatment. (**d**) 0.015 silane coupling agent with an average cluster size of <~30 µm, (**e**) 0.020 silane coupling agent with an average cluster size of <~25 µm, and (**f**) 0.025 silane coupling agent with an average cluster size of ~25–30 µm.

The SEM micrograph images shown in Figure 6 represent the cluster size of pristine and surface treated BaTiO_3_ particles, respectively. From the distribution plots shown in Figure 7, it can be seen that the cluster size reduces after surface modification with a silane coupling agent, i.e., from ~60 µm to around ~30 µm. A summary of the average particle diameters is provided in Table 5. The image in Figure 6B did undergo some charging due to the adsorbed insulative organofunctional silane layer and as result had to be taken at lower EHT voltage < 5 kV (i.e., lower gun voltage). Figure 6A, however, contains no such insulative layer and hence could be observed at higher EHT voltage > 7 kV.

**Figure 8:**
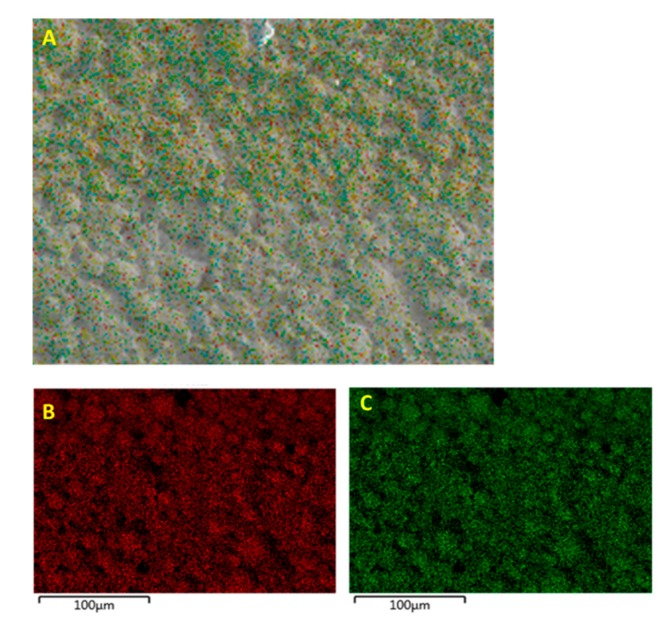
EDS micrograph images of the fractured surface of the pristine (not surface treated) BT(0.5)/epoxy composite film where (**A**) overall surface of the composite, (**B**) distribution of Ba and (**C**) Ti within the overlaying epoxy matrix.

**Figure 9:**
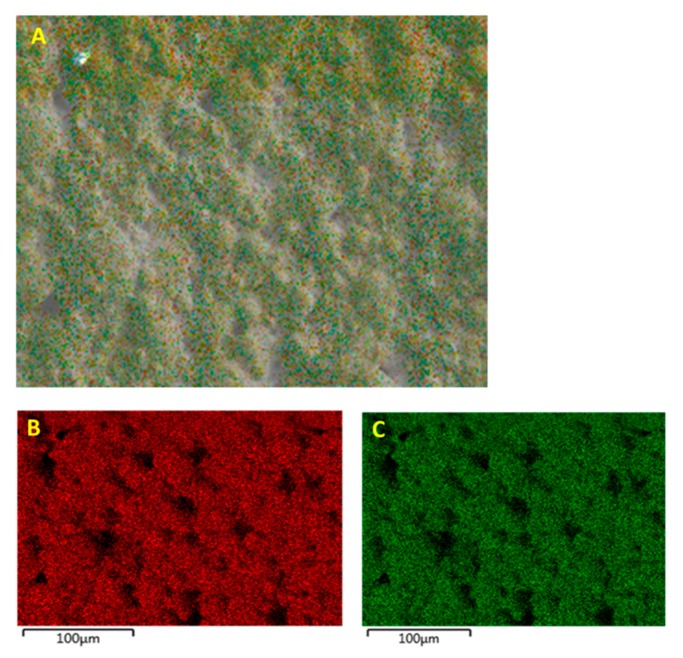
EDS micrograph images of the fractured surface of the SCA 0.010 surface treated BT (0.5)—Epoxy composite film where (**A**) is the overall surface of the composite, (**B**) distribution of Ba and (**C**) Ti particles within the overlaying epoxy matrix.

The EDS micrograph images were taken for samples with 0.5 volume fraction of BaTiO_3_ as the reference because for our measurements, they represent the best properties. The EDS micrographs showing the fractured surface of the BT (0.5)—epoxy composite that were not surface treated is shown in Figure 8. From the images one can see that there are regions of aggregations of the BaTiO_3_ particles caused by one of two reasons or both, (i) the nanoparticles themselves formed clusters to reduce their surface energy and/or (ii) the incompatibility between the inorganic ceramic filler BaTiO_3_ and the organic polymer epoxy resin. It is quite evident that agglomerations are undesirable due to increased difficulty in processing, formation of voids and thereby leading to ineffective poling. Surface modification of these composites with a silane coupling agent can greatly improve the overall distribution of the inorganic filler particles as can be seen from the EDS micrograph images in Figure 9. Compared to the composites that were not surface treated in Figure 8, there is a greater “density” of particles, which coincidentally increases the total surface area creating more interfacial regions between BaTiO_3_ and the epoxy matrix. The enhanced dispersion leads to more effective poling as the applied electric field will have influenced more ferroelectric particles within the matrix.

**Figure 10:**
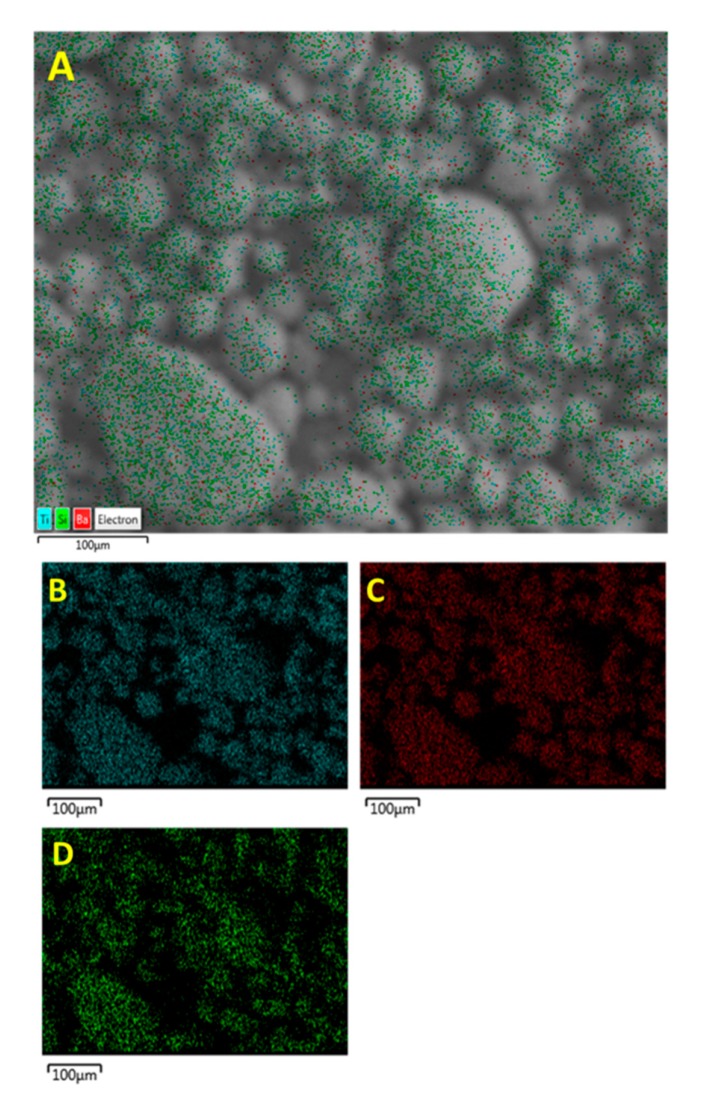
EDS micrograph images of SCA 0.015 surface modified BaTiO_3_ powder showing (**A**) the EDS layered image and the distribution of (**B**) Ti, (**C**) Ba and (**D**) Si particles.

In Figure 10, it can be seen that Si has the same particle distribution as Ba and Ti, which is indicative of the successful modification of the surface of the BaTiO_3_ nanoparticle. While there is a 1:1 correspondence between (B) and (C) as they from the same compound i.e., BaTiO_3_, we see that the Si atoms do not share the same 1:1 correspondence. This could be attributed to the fact that the volume fraction of silane coupling agent i.e., 0.015 was not sufficient to generate maximum number of reactions.

**Figure 11:**
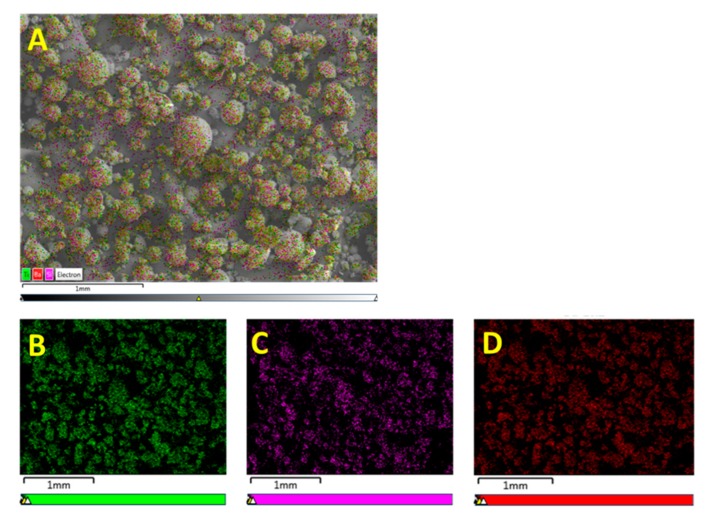
EDS micrograph images of BaTiO_3_ nanoparticles that were surface treated using SCA 0.020 taken at a scale of 100 µm. It can be seen that Si was successfully coated on to the surface of the BaTiO_3_ nanoparticles as seen in (**A**). The images in (**B**–**D**) represent the particle distributions of Ba, Ti and Si, respectively. Compared to the samples surface treated using SCA 0.015, we notice that there is a greater correspondence between the Ba atoms in (**B**) and the Si atoms in (**D**).

**Figure 12:**
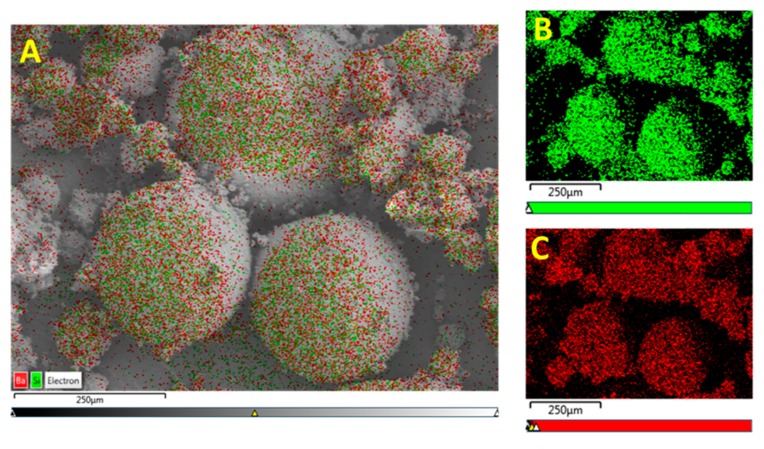
(**A**) EDS micrograph images of the BaTiO_3_ nanoparticles that were surface treated using SCA 0.025. The green color depicts the (**B**) particle distribution of silicon atoms and the red denotes (**C**) the barium atoms.

**Figure 13:**
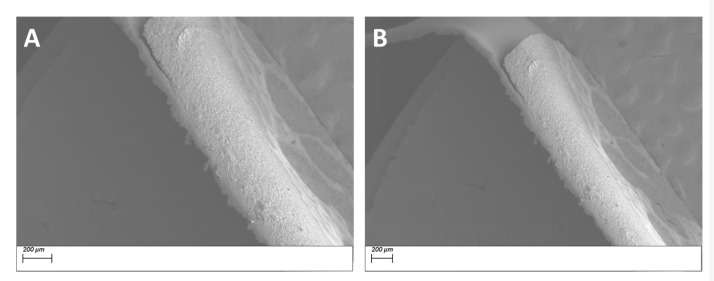
SEM micrograph images of the cross-section of the fractured surface of the composite that was prepared using 0.5 volume fraction 0.020 silane coupling agent BaTiO_3_ nanoparticles at (**A**) 129 X and (**B**) 93 X.

From the EDS micrograph images shown in Figure 11 for the 0.020 silane coupling agent surface modified BaTiO_3_ powders one can see that the particle distribution of Ba, Ti and Si are almost identical. Also from the EDS layered image shown in Figure 10A, one can see that Si sits on the surface of the BaTiO_3_ particles, indicating that successful surface modification occurred. For composites prepared using SCA 0.025 volume fraction of coupling agent shown in Figure 12 we see a greater correspondence with the Ba and Si atoms, which can be attributed to the increased number of reactions between the BaTiO_3_ nanoparticle and the silane coupling agent. This almost represents a case of saturated reactions wherein further increases in concentration will yield no further results. In Figure 13, a SEM micrograph image of the cross-section of the fractured surface of the composite that was prepared using 0.5 volume fraction 0.020 silane coupling agent BaTiO_3_ nanoparticles at (A) 129 X and (B) 93 X. The images show that surface treating the composite with a coupling agent results in good adhesion between the composite film and the stainless steel substrate.

**Figure 14:**
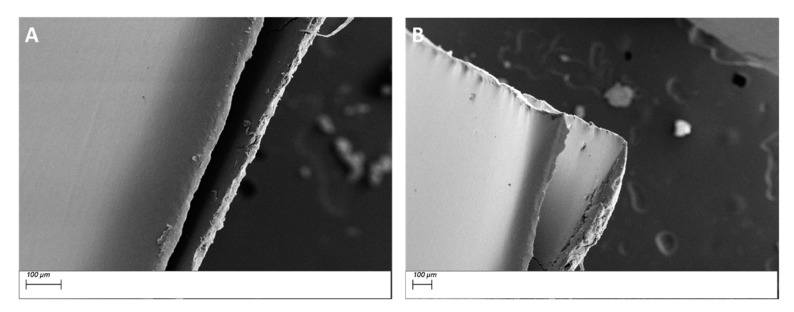
SEM micrograph images of (**A**) SCA 0.025 BT (0.5)/epoxy composite at 280 X and (**B**) SCA 0.025 BT (0.6)/epoxy at 151 X; both showing significant delamination of the film from the composite. It is interesting to note that there is no cracking on the composite film, it delaminates as a whole.

From the cross-section images shown in Figure 14, for the BaTiO_3_/epoxy composites fabricated using 0.025 SCA at 50% volume fraction of BaTiO_3_ the observations indicate severe delamination of the film from the substrate. It is important to note that this delamination is not caused by cracking of the film, instead the film is intact, and peels off as a whole.

### 3.2. Piezoelectr Strain Characteristics

#### 3.2.1. Piezoelectric Strain Coefficients—Surface Treatment with Ethanol—Datum

**Figure 15:**
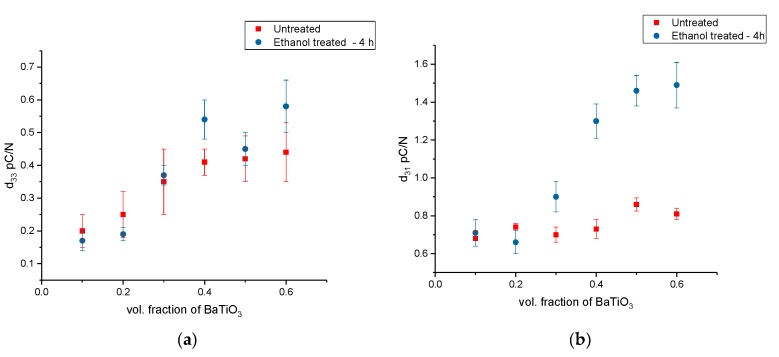
(**a**) The piezoelectric strain coefficient, d_33_ for the BaTiO_3_-epoxy composite fabricated using BaTiO_3_ nanoparticles that were surface modified using ethanol for 4 h is plotted as a function of volume fraction of BaTiO_3_. The maximum value is 0.58 pC/N at 0.6 vol. fraction of BaTiO_3_. (**b**) The piezoelectric strain coefficient, d_31_ for the BaTiO_3_-epoxy composite fabricated using BaTiO_3_ nanoparticles that were surface modified using ethanol for 4 h is plotted as a function of volume fraction of BaTiO_3_. The maximum value is 1.49 pC/N at 0.6 volume fraction of BaTiO_3_.

**Figure 16:**
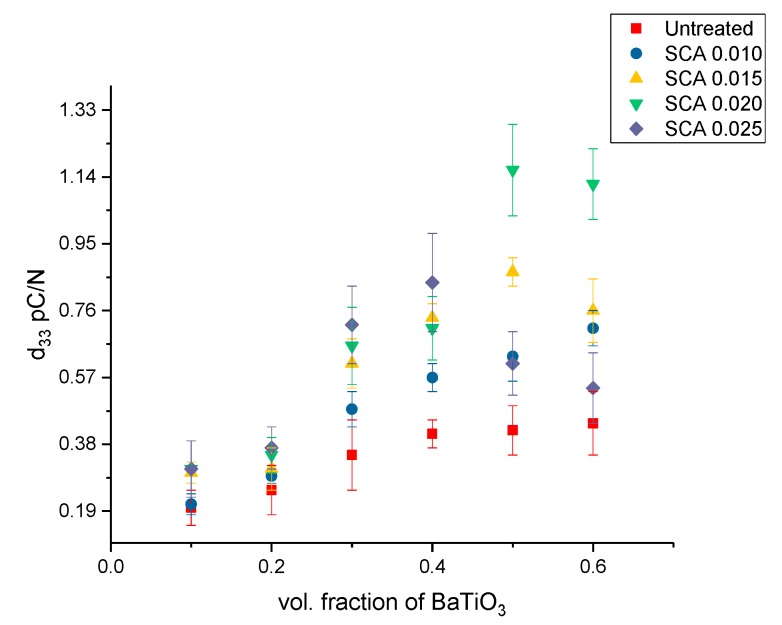
The piezoelectric strain coefficient, d33, for the BaTiO_3_-epoxy composite that were fabricated using BaTiO_3_ nanoparticles which were untreated, surface modified by 0.01 SCA, 0.015 SCA, 0.02 SCA and 0.025 SCA. The maximum d33 values are 0.44, 0.71, 0.87, 1.16 and 0.84 pC/N at volume fractions of 0.6, 0.6, 0.5, 0.5 and 0.4, respectively.

The BaTiO_3_-epoxy composites were fabricated using BaTiO_3_ nanoparticles that were surface modified using ethanol for 4 h. The sonication of these particles in ethanol improved their dispersion and led to improved piezoelectric strain coefficient values compared to pristine BaTiO_3_-epoxy composites, as can be seen in Figure 15 and Figure 16.

It can be seen in Figure 15 that the piezoelectric properties *d*_33_ and *d*_31_ increase with increasing volume fraction of the BaTiO_3_ nanoparticles within the composite. Ethanol only acts as a dispersant, i.e., it can only improve dispersion of the fillers and not act as a binding or molecular bridge between fillers and polymers [76,77,78,79], however the ethanol does aid in better dispersion of the particles within the composite, and as previously mentioned is due to electrostatic stabilization.

#### 3.2.2. Piezoelectric Strain Coefficients—Surface Treatment with Silane Coupling Agent (KH 560)

A comparison plot showing the variation in the *d*_33_ across all the composites is shown in Figure 16. Largely, the *d*_33_ shows a similar trend culminating at 50% BaTiO_3_ except for the composites surface treated using SCA 0.01 where it peaks at 60%. Followed by initially surface treating these composites with KH-560 at 0.01 the concentration was subsequently increased to SCA 0.015, SCA 0.020 and SCA 0.025. By varying the concentration of the coupling agent during surface treatment it enables us to gain a broader understanding of its interaction with BaTiO_3_ nanoparticles.

**Figure 17:**
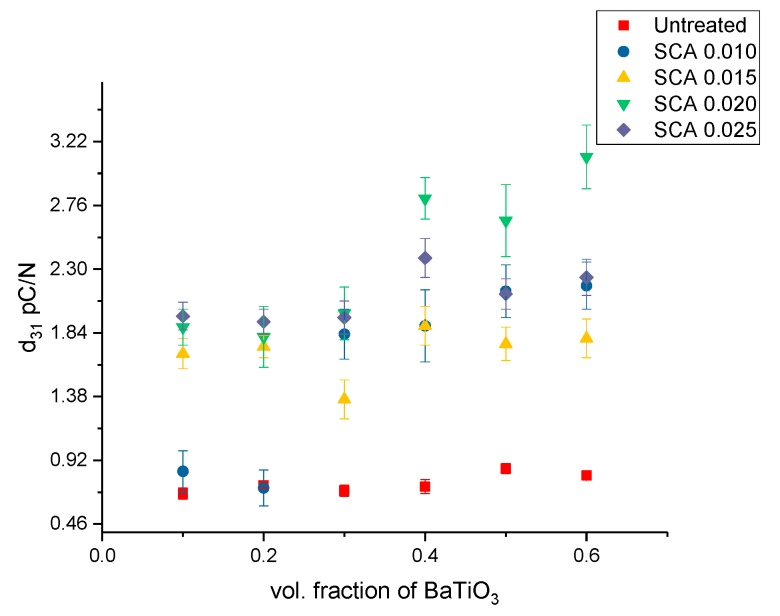
The piezoelectric strain coefficient, *d*_31_, for the BaTiO_3_-epoxy composite that were fabricated using BaTiO_3_ nanoparticles which were untreated, surface modified by 0.01 SCA, 0.015 SCA, 0.02 SCA and 0.025% SCA. The maximum *d*_31_ values are 0.86, 2.18, 1.89, 3.11 and 2.38 pC/N at volume fractions of 0.6, 0.6, 0.5, 0.5 and 0.4, respectively.

The piezoelectric strain coefficients for sample surface treated using SCA 0.010, SCA 0.015, SCA 0.020 and SCA 0.025 is shown in Figure 16 and Figure 17. The BaTiO_3_ nanoparticles were then surface modified using silane coupling agent, KH-560 at a volume fraction of 0.01. As can be seen from Figure 9 this created more number of interfacial regions between BaTiO_3_ and epoxy creating a more uniform and homogeneous dispersion of nanoparticles within the matrix. The observations shown in these figures exhibit a similar trend where the value of *d*_33_ increase with increasing concentration of BaTiO_3_. This increase in *d*_33_ values as volume of the ferroelectric particle is increased is consistent with the observations of several researchers [31,80,81,82,83,84,85,86,87]. However, in these composites there seems to be a drop in the value at 0.6 volume fraction. This drop in value could be due to the formation of voids at higher volume fractions thereby increasing the overall porosity of the composite and making them more difficult to pole. Or, the decrease in longitudinal piezoelectric strain coefficient could be due to the delamination of the composite from the substrate, leading to ineffective poling thereby weakening its form and function.

The composites that were surface modified using SCA 0.025 suffered from delamination at higher volume fractions viz. 50% and 60% as shown in Figure 14. In these composites, quite unusually, the composites were peeling off of the substrate. At higher concentrations of coupling agent i.e., 0.025, there are a higher number of covalent bonds formed especially at higher volume fraction of BaTiO_3_ nanoparticles i.e., 0.5 or higher. As a result there are increased number of BaTiO_3_-epoxy-silane coupling agent bonds as can be seen from the EDS images above, which lead to improved cohesion of the composite but at the cost of weakened substrate adhesion, i.e., the cohesive strength of the composite outweighs the adhesive strength resulting in complete delamination of the composite but remarkably no cracking of the composite occurs.

For the composites that were untreated, the *d*_31_ at 50% volume fraction of BaTiO_3_ was recorded to be 0.86 pC/N and the *d*_33_ is 0.42 pC/N, which is more than double. The magnitude of the *d*_31_ values is considerably higher than the *d*_33_ values owing to the higher strain in the direction along the 1-axis as compared to the strain in the 3-axis which is consistent with other reports [37,88]. The general trend is as expected, the *d*_3_ value increases with increasing volume fraction of the BaTiO_3_ nanoparticle. Similarly, for the SCA 0.01 surface treated samples, at 60% volume fraction of BaTiO_3_ the *d*_33_ is 0.71 pC/N while the *d*_31_ is 2.18 pC/N, which is almost 4 times in magnitude.

The *d*_31_ values increase once they have been surface treated, which could be ascribed to better particle distribution leading to more homogeneous dispersion [24,71]. Ramesh et al. [52] reported that the surface modified BaTiO_3_ composites demonstrate enhanced mechanical response due to increased cross link density in the epoxy network resulting in the closer connectivity of particle and the matrix network which could also be attributed to the increased piezoelectric coefficient. The maximum *d*_31_ value occurs for the BaTiO_3_/epoxy composite that were surface treated using a 0.020 concentration of silane coupling agent KH-560. The longitudinal piezoelectric strain coefficients are along the same magnitude of those reported by others [9,42,43], while some workers values are slightly higher due to the piezoelectric nature of the matrix (PVDF) that was not the case for the work herein. It is well known that the crystal structure of BTO powder changes with its size, where at room temperature tetragonality decreases as BTO particle mean diameter decreases up to ~0.3 µm, and below this diameter it decreases more to ~0.1 µm, where below this, cubic and pseudo-cubic structures exist [41,42,44,89], which results in substantial decreases in piezoelectric coefficients below mean diameters of 0.3 µm. In addition, the coating of the nano-particles with an insulative layer enhances the bond between the particle and the matrix, which ultimately enhances the matrix’s ability to carry the charge from the polarization process to the particle surface by overcoming the surface resistance and incompatibility between the matrix material and the BTO particles.

### 3.3. Resistivity

#### 3.3.1. Resistivity—Surface Treatment with Ethanol—Datum

**Figure 18:**
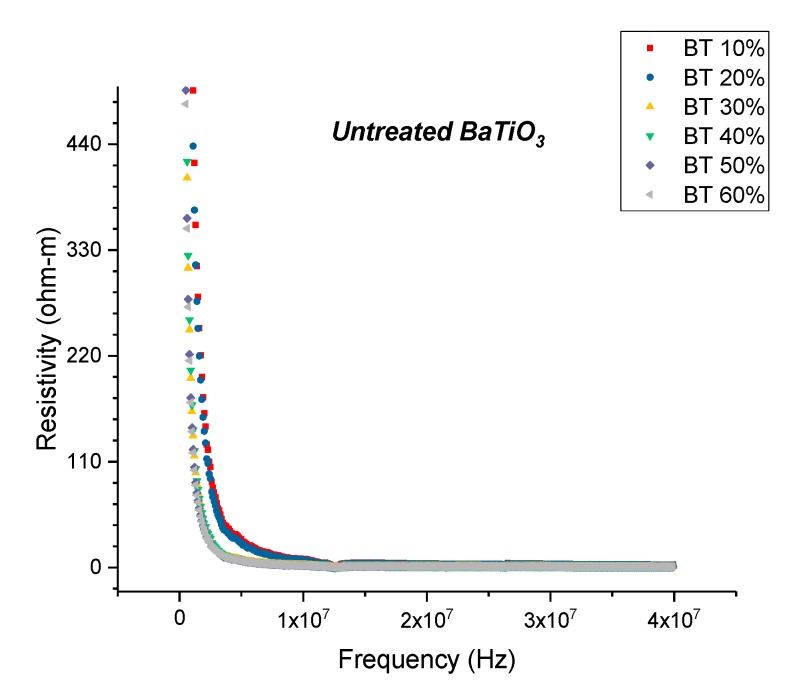
The resistivity of the BaTiO_3_-epoxy composites that were fabricated using BaTiO_3_ nanoparticles which were not surface treated (pristine) is plotted as a function of frequency. The maximum resistivity recorded at 20 MHz was 4.20 ohm-m which corresponds to the composites containing 10% of BaTiO_3_.

The resistivity and conductivity measurements were recorded using an HP 4194A Impedance Analyzer over a wide frequency spectrum i.e., between 2000 Hz and 40 MHz to gather insight into the resistive behavior of these composites. In Figure 18: The resistivity of the BaTiO_3_-epoxy composites that were fabricated using BaTiO_3_ nanoparticles which were not surface treated (pristine) is plotted as a function of frequency. The maximum resistivity recorded at 20 MHz was 4.20 ohm-m which corresponds to the composites containing 10% of BaTiO_3_.

#### 3.3.2. Resistivity—Surface Treatment with Ethanol—Datum

The resistivity measurements as a function of frequency is shown in Figure 18 for composites that were fabricated with pristine or non-surface treated BaTiO_3_. At 20 MHz the maximum resistivity was recorded to be 4.20 ohm-m at 0.1 volume fraction of BaTiO_3_. With increasing volume fraction of BaTiO_3_ the value of resistivity began to decrease, and the lowest recorded resistivity at 20 MHz was found to be 1.02 ohm-m at 0.6 volume fraction.

**Figure 19:**
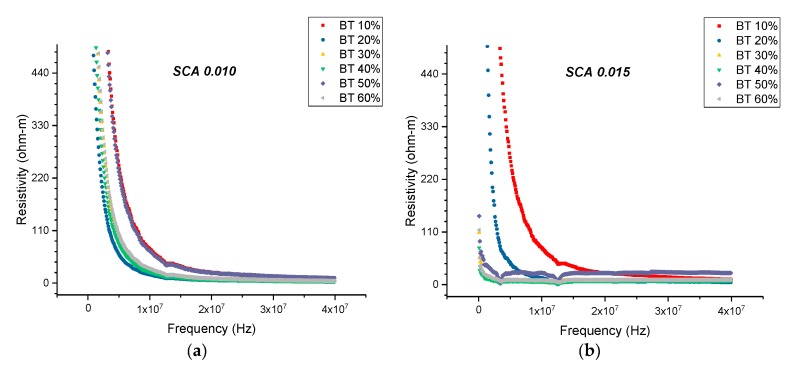
(**a**) The resistivity of the BaTiO_3_-epoxy composite that were fabricated using BaTiO_3_ nanoparticles, which were surface-modified using 0.010 SCA, which is plotted as a function of frequency. The maximum resistivity recorded at 20 MHz was 22.08 ohm-m which corresponds to the composites containing 0.1 of BaTiO_3_. (**b**) The resistivity of the BaTiO_3_-epoxy composite that were fabricated using BaTiO_3_ nanoparticles, surface-modified using 0.015 SCA, is plotted as a function of frequency. The maximum resistivity recorded at 20 MHz was 24.26 ohm-m which corresponds to the composites containing 0.5 of BaTiO_3_.

At 20 MHz, the maximum resistivity was recorded to be 24.26 ohm-m for the sample containing 0.5 of BaTiO_3_ and surface treated using SCA 0.015, as shown in Figure 19. For the same volume fraction of BaTiO_3_ but for the samples surface treated at SCA 0.020 and 0.025, the resistivity was recorded to be 39.76 ohm-m, and 16.81 ohm-m, respectively. The initial increase in resistivity can be attributed to the better particle-polymer adhesion, thereby, reducing conduction loss. Surface treating the sample substitutes the hydroxyl groups, present on the surface of the BaTiO_3_ nanoparticle, with hydroxyl group on an organofunctional silane. It replaces conductive hydroxyl groups with an insulating polymer, leading to improved adhesion with the epoxy matrix and reducing the electrical conductivity at the interface. However, when the coupling agent concentration is increased to 0.025, there is an increase in film delamination, leading to inefficient poling and increased conduction losses.

**Figure 20:**
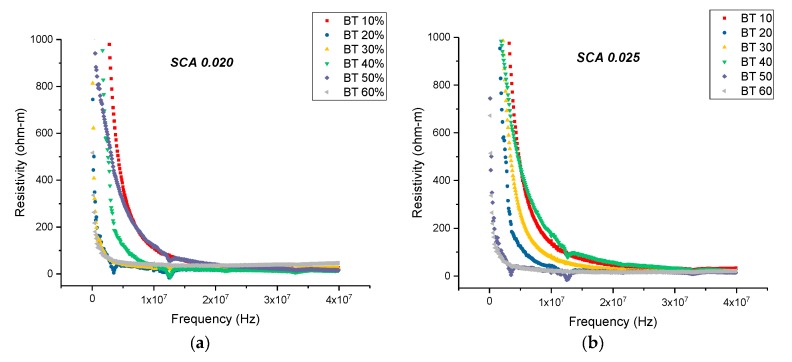
(**a**) The resistivity of the BaTiO_3_-epoxy composite that were fabricated using BaTiO_3_ nanoparticles. These nanoparticles were surface modified using 0.02 SCA is plotted as a function of frequency. The maximum resistivity recorded at 20 MHz was 39.76 ohm-m which corresponds to the composites containing 0.5 of BaTiO_3_. (**b**) The resistivity of the BaTiO_3_-epoxy composite that were fabricated using BaTiO_3_ nanoparticles, which were surface modified using 0.02 SCA is plotted as a function of frequency. The maximum resistivity recorded at 20 MHz was 58.22 ohm-m which corresponds to the composites containing 0.4 of BaTiO_3_.

There is an increase in resistivity by ~73% at 20 MHz between the composites prepared using SCA 0.015 and SCA 0.02 at 0.5 volume fraction of BaTiO_3_ as shown in Figure 19 and Figure 20. In Figure 20, the resistivity of the BaTiO_3_-epoxy composite that were fabricated using BaTiO_3_ nanoparticles, which were surface-modified using 0.02 SCA and 0.025 SCA, are plotted as a function of frequency. The maximum resistivity recorded at 20 MHz was 39.76 ohm-m and 58.22 ohm-m, respectively, for composites containing 0.5 of BaTiO_3_. The resistivity of each sample increases as a function of coupling agent concentration at 20 MHz.

**Figure 21:**
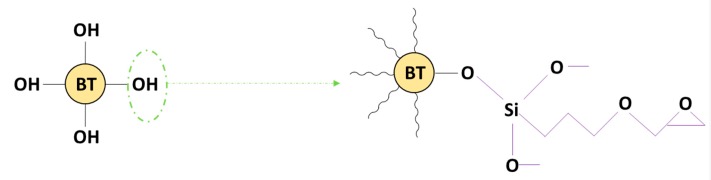
Substitution of the hydroxyl groups due to the surface modification with the silane coupling agent. This leads to a decrease in conductivity as there is no more water adsorbed on the surface of the BaTiO_3_ nanoparticle.

The pristine or untreated BaTiO_3_ due to its hydrophilic nature tends to adsorb water onto its surface [24,25]. The surface modification leads to the substitution of the hydroxyl (-OH-) groups on the surface of the BaTiO_3_ with a modified surface chemistry (O-Si-) as shown in Figure 21. Therefore, when the concentration of coupling agent is increased, such reactions may occur, thereby, lowering the conductivity values [32,90].

## 4. Conclusions

Two-phase BaTiO_3_/epoxy composite films were fabricated where the volume fraction of the BaTiO_3_ nanoparticle was varied from 0.1 to 0.6. In order to ensure uniform dispersion of the filler nanoparticles within the epoxy matrix the BaTiO_3_ was surface treated using; (i) ethanol and (ii) silane coupling agent, KH-560. The piezoelectric strain coefficients and resistivity values were measured, and calculated, respectively. Different concentrations (*v*/*v*) of the coupling agent were used for the surface modification of BaTiO_3_; 0.01, 0.015, 0.02 and 0.025.

When the concentration of the coupling agent was increased, an increase in the piezoelectric strain coefficient values of the BaTiO_3_/epoxy composite was observed. This could be attributed to better dispersion of the BaTiO_3_ nanoparticles as observed using the EDS and SEM micrograph images. The SEM micrograph images helped provide a particle size analysis that showed that the mean diameter of the nano-clusters reduced in size with increasing concentration of silane coupling agent. The presence of silicon molecules that were adsorbed on to the surface of the BaTiO_3_ nanoparticle were observed using the EDS micrograph images, which also showed a reduction in the aggregate size, and led to better particle distribution. The improved adhesion between the polymer-ceramic interface led to higher interfacial polarization within the composite material, and as result led to increased piezoelectric strain values.

The resistivity values were measured for each concentration of the silane coupling agent, and it was found that the resistivity increased as a function of coupling agent concentration. The maximum conductivity was observed for untreated, SCA 0.01, SCA 0.015, SCA 0.020, and SCA 0.025 treated BaTiO_3_ nanoparticles at 20 MHz. This could be ascribed to the substitution of the hydroxyl (-OH-) groups on the surface of the BaTiO_3_ nanoparticle with that of the coupling agent. By eliminating the formation of adsorbed water on the surface of the particle, the conductivity of the system was gradually reduced by increasing the amount of the coupling agent.

## Figures and Tables

**Table 1:** Physical properties of BaTiO_3_ [11,68].

Barium Titanate Nano-Powder	
**Property**	
Density (g/cm^3^)	6.08
Mean Diameter *	~100 nm
Curie Point (°C)	130 °C
Dielectric constant	150 [69]
*d*_33_ (pC/N)	~85.6 (crystal)/~191 (ceramic)

* As received from manufacturer.

**Table 2:** Physical, dielectric and piezoelectric properties of dyglycidyl ether of bisphenol A (DGEBA) epoxy [67].

Property	
Dielectric Constant	2.9–3.0 *
Dielectric dissipation	~0.02–0.04
Electromechanical coupling factor, *k*_33_	--
Piezoelectric strain coefficient, *d*_33_	--
Density	1.16 g/cm^3^
Electrical Resistivity	0.15 @ 1 kHz

* Experimental values.

**Table 3:** Physical properties of 3-glycidyloxypropyltrimethoxysilane (KH560).

Property	
Density	1.07 g/mL
Boiling point	120 °C/2 mmHg(lit.)

**Table 4:** Varying concentration of silane coupling agent for surface treatment of BaTiO_3_ nanoparticle.

KH-560	Set 1	Set 2	Set 3	Set 4
**Concentration**	0.01	0.015	0.020	0.025

**Table 5:** Table summarizing the average particle size diameter of the nano-clusters. Surface treatment helps in reducing the cluster size and improves with increasing concentration of coupling agent.

Sample Set.	Average Particle Diameter (Nano-Clusters)
Non-surface Treated BaTiO_3_	~60 µm
Ethanol Surface Treated (4 h)	~40 µm
Silane Coupling Agent–0.010	~30 µm
Silane Coupling Agent–0.015	~<30 µm
Silane Coupling Agent–0.020	~25 µm
Silane Coupling Agent–0.025	~28 µm
